# Expression of Stem Cell Niche-Related Biomarkers at the Base of the Human Tricuspid Valve

**DOI:** 10.1089/scd.2022.0253

**Published:** 2023-03-03

**Authors:** Linnéa Sjölin, Marianne Jonsson, Charlotta Orback, Anders Oldfors, Anders Jeppsson, Jane Synnergren, Victoria Rotter Sopasakis, Kristina Vukusic

**Affiliations:** ^1^ Department of Laboratory Medicine, Institute of Biomedicine, and Institute of Medicine, Sahlgrenska Academy, University of Gothenburg, Gothenburg, Sweden.; ^2^ Department of Clinical Chemistry, Sahlgrenska University Hospital, Gothenburg, Sweden.; ^3^ Department of Pathology, and Sahlgrenska University Hospital, Gothenburg, Sweden.; ^4^ Department of Cardiothoracic Surgery, Sahlgrenska University Hospital, Gothenburg, Sweden.; ^5^ Department of Molecular and Clinical Medicine, Institute of Medicine, Sahlgrenska Academy, University of Gothenburg, Gothenburg, Sweden.; ^6^ Department of Biology and Bioinformatics, School of Bioscience, University of Skövde, Skövde, Sweden.

**Keywords:** heart, stem cell niche, atrioventricular junction, hypoxia, cardiomyocyte proliferation

## Abstract

Stem cell niches have been thoroughly investigated in tissue with high regenerative capacity but not in tissues where cell turnover is slow, such as the human heart. The left AtrioVentricular junction (AVj), the base of the mitral valve, has previously been proposed as a niche region for cardiac progenitors in the adult human heart. In the present study, we explore the right side of the human heart, the base of the tricuspid valve, to investigate the potential of this region as a progenitor niche. Paired biopsies from explanted human hearts were collected from multi-organ donors (*N* = 12). The lateral side of the AVj, right atria (RA), and right ventricle (RV) were compared for the expression of stem cell niche-related biomarkers using RNA sequencing. Gene expression data indicated upregulation of genes related to embryonic development and extracellular matrix (ECM) composition in the proposed niche region, that is, the AVj. In addition, immunohistochemistry showed high expression of the fetal cardiac markers MDR1, SSEA4, and WT1 within the same region. Nuclear expression of HIF1α was detected suggesting hypoxia. Rare cells were found with the co-staining of the proliferation marker PCNA and Ki67 with cardiomyocyte nuclei marker PCM1 and cardiac Troponin T (cTnT), indicating proliferation of small cardiomyocytes. WT1+/cTnT+ and SSEA4+/cTnT+ cells were also found, suggesting cardiomyocyte-specific progenitors. The expression of the stem cell markers gradually decreased with distance from the tricuspid valve. No expression of these markers was observed in the RV tissue. In summary, the base of the tricuspid valve is an ECM-rich region containing cells with expression of several stem cell niche-associated markers. Co-expression of stem cell markers with cTnT indicates cardiomyocyte-specific progenitors. We previously reported similar data from the base of the mitral valve and thus propose that human adult cardiomyocyte progenitors reside around both atrioventricular valves.

## Introduction

To maintain tissue homeostasis and initiate tissue repair, generating new cells is crucial. The regeneration is potent enough to heal small injuries, but for cardiomyocytes, damage often results in formation of scar tissue and permanent loss of function.

Throughout life, new cardiomyocytes are generated in the human heart with a low turnover rate, roughly 1% per year [1,2]. How these new cardiomyocytes are formed and where they originate from is not fully understood. In other tissues, new cells are produced by activation, proliferation, and differentiation of stem cells that reside in stem cell niches [3]. Adult stem cells are quiescent and slow cycling in the niches. Therefore, the niches have been identified by DNA labeling with 5-bromo-2-deoxyuridine (BrdU). These niches are hypoxic anatomical structures, extensively studied in tissues with rapid cell turnover such as the skin [4], liver [5], and cornea [6]. The low oxygen tension is one of the hallmarks of the hematopoietic niches where hypoxia-inducible factor 1 (HIF1α) plays a role in cell cycle quiescence [7]. A specific composition of extracellular matrix (ECM) modulates the proliferation, self-renewal, or differentiation of stem cells in the niche [8,9].

In the field of cardiomyocyte regeneration, there are currently three main theories of the origins of new cardiomyocytes: exogenous stem cells [10], endogenous stem cells [11], or dedifferentiation of pre-existing cardiomyocytes [12,13]. While most focus has shifted toward dedifferentiation of pre-existing cardiomyocytes, evidence has both been presented and challenged for each of them.

BrdU labeling has been used for identification of stem cell niches in tissues with low regenerative potential, such as the cartilage [14] and brain [15]. In the murine heart, niches were described as nests of BrdU+ cells, with no specific anatomical structure [16]. We combined BrdU labeling with physical exercise for activation of stem cells in a rat model and observed slow cycling BrdU+ cells surrounding the tricuspid and mitral valves in an anatomical structure called the AtrioVentricular junction (AVj) [17]. In addition, we found expression of the stem cell markers MDR1 and Sca1. In a follow-up study on the human left AVj, the base of the mitral valve, we observed expression of biomarkers associated with hypoxia, stem cells, proliferation, and migration, showing features of a progenitor niche [18].

In the present study, we explored the human right AVj, the base of the tricuspid valve, using RNA sequencing and immunohistochemistry (IHC) for investigation of stem cell niche properties. Early fetal cardiac-specific stem cell markers SSEA4 [19,20] and WT1 [21] were of particular interest since cell populations expressing these markers have been described as a source for cardiac regeneration. A characteristic common for many stem cells is that they can exclude toxic substances. Side population progenitor cells, expressing an efflux protein MDR1, have been identified in human cardiac tissue [22–24]. Proliferation markers Proliferating Cell Nuclear Antigen (PCNA) and Ki67 combined with cardiomyocyte-specific nuclei marker PCM1 [2,25] and cardiac Troponin T (cTnT) were used to investigate new formation of cardiomyocytes.

## Materials and Methods

### Human cardiac biopsies

**FIG. 1.**
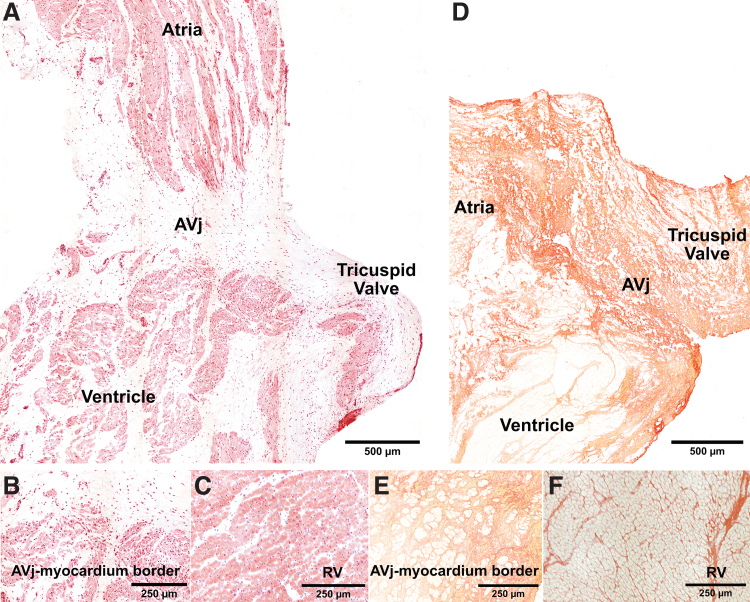
Histology of the human hearts showcasing the anatomical regions of the tissue samples analyzed during this study. **(A)** Histology of the right AVj biopsy from a representative organ donor, stained with hematoxylin–eosin. Parts of the RA and RV are included as well as the tricuspid valve. The suggested stem cell niche region is in the connective tissue between the ventricle and atria. **(B)** Enlargement of the border to ventricular myocardium to the AVj displaying small cardiomyocytes compared with the larger cardiomyocytes **(C)** in a representative RV biopsy. **(D)** Picric Sirius red staining of the AVj showing collagen content in *red*. The annulus pulposus and the tricuspid valve are collagen rich, whereas the ventricle, atria, and the epicardiac adipose tissue show less collagen content. **(E)** Enlargement of small cardiomyocytes (*yellow*) embedded in collagen (*red*) in the myocardium bordering to the AVj showing higher collagen content in the AVj region compared with the RV tissue **(F)**. AVj, AtrioVentricular junction; RA, right atria; RV, right ventricle.

This study was approved by the research ethics board at the Sahlgrenska Academy, University of Gothenburg, Sweden, following the Declaration of Helsinki. Biopsies were harvested from explanted hearts from male and female multi-organ donors (*N* = 12), 19–75 years. The hearts used were not suitable for heart transplantation but explanted for aortic and pulmonary valve homograft procurement. All had documentation of consent from the donor, stating that their organs can be used for other medical purposes than transplantation. Clinical background is summarized in Supplementary Table S1. Biopsies for the present study were collected from three locations from each heart: (1) right AVj located at the base of the tricuspid valve, (2) lateral side of the right atria (RA), and (3) lateral side of right ventricular myocardium (right ventricle [RV]) (Fig. 1). The AVj biopsies include small parts of atria and ventricle, as shown in Fig. 1A and D.

### Histology

Biopsies from the AVj and RV were embedded in Tragacanth mounting medium (Histolab Products AB, Gothenburg, Sweden), frozen in liquid nitrogen, and stored in a −80°C. The biopsies were sliced into 7 μm sections and stained with hematoxylin–eosin and Picric Sirius red for histology.

### RNA extraction

Tissue for gene analyses was placed in RNAlater (Qiagen, Valencia, CA) and stored in −80°C. Thirty to 100 mg of tissue per sample was homogenized in a TissueLyser LT and lysed with QIAzol (Qiagen). 1-Bromo-3-chloropropane was used instead of chloroform since it is less toxic. The water phase was further purified with the RNeasy Mini Kit in the QIAcube (Qiagen) according to the protocol. DNase1 was used to remove genomic DNA. The RNA was eluted in RNase free water.

### RNA sequencing analysis

Library construction was performed using Illumina TruSeq stranded total RNA with Illumina Ribozero method. Clustering was carried out by “cBot,” and samples were sequenced on NovaSeq6000 (NovaSeq Control Software 1.6.0/RTA v3.4.4) with a 2 × 51 setup using “NovaSeqXp” workflow in “S1” mode flow cell. The Bcl to FastQ conversion was performed using bcl2fastq_v2.19.1.403 from the CASAVA software suite. The quality scale used was Sanger/phred33/Illumina 1.8+. Processing of FASTQ files was carried out by the SciLifeLab National Genomics Infrastructure at the Uppsala Multidisciplinary Center for Advanced Computational Science, Sweden. Sequenced reads were quality controlled with the FastQC software and preprocessed with Trim Galore.

### Statistics and bioinformatics

The raw data were aligned with the human GRCh38.107 reference library from the Ensembl genome browser (https://www.ensembl.org/Homo_sapiens/Info/Index), and the resulting BAM files were used for bioinformatics analysis. Statistical and bioinformatics analysis and gene set enrichment analysis (GSEA) of the gene data were performed with Qlucore Omics Explorer 3.8 (Qlucore AB, Lund, Sweden). The Molecular Signatures Database (https://www.gsea-msigdb.org/gsea/msigdb/index.jsp) was used to obtain gene sets for the GSEA.

The Qlucore Omics Explorer software utilizes trimmed mean of *M* values normalization and genomic feature length normalization and performs log2 transformation of the data before analysis. Transcripts with counts ≥10 were included in the analysis. Paired group comparison of five individuals was performed, and levels of significance for differences between group means were determined with t-test or two-way analysis of variance, followed by Tukey's multiple comparison tests. A false discovery rate-adjusted *P* value (*q* value) <0.05 was considered significant.

### Immunohistochemistry

The frozen tissue sections were fixed in −20°C acetone for 10 min and washed in phosphate-buffered saline (PBS). Background was blocked with 2% bovine serum albumin, 0.3% Triton-X100, and 5% goat serum (Invitrogen, Carlsbad, CA) in PBS for 30 min at room temperature (RT). Three primary antibodies were combined in each protocol, diluted according to Supplementary Table S2. Primary antibodies were incubated at 4°C overnight. The sections were washed, and the following secondary antibodies were added for 1 h at RT: goat anti-mouse Alexa Fluor 546, goat anti-rabbit Alexa Fluor 546, or goat anti-rabbit Alexa Fluor 647 (Invitrogen). To enable the use of two mouse primary antibodies, the cTnT antibody was conjugated with Alexa 488 fluorochrome using Zenon Kit (Invitrogen). The samples were fixed using Histofix (Histolab, Gothenburg) for 15 min, washed, and mounted with prolong gold antifade reagent with nuclei staining 4′,6-diamidino-2-phenylindole (DAPI) (Invitrogen). Corresponding isotype controls for primary antibodies and did not show specific staining.

### Image analysis

An ECLIPSE Ti inverted microscope (Nikon Corporation, Tokyo, Japan) was used for brightfield microscopy for histology analysis with a Nikon DS-2Mv camera and fluorescence for IHC analysis with an Andor's Zyla camera.

To ensure that all nuclei were captured in focus, the images were acquired at three different depths (Z-levels). Large images were produced by stitching together 7 × 7 photos of fields with 20 × objective to cover the AVj region. Generally, four channels (blue DAPI, green Alexa 488, yellow Alexa 546, and red Alexa 647) were acquired. Expression of biomarkers was analyzed in large composite photos using the software ImageJ (v1.53e; Fiji distribution). The ranges for each channel were set to eliminate most background based on isotype controls. The large images were cropped to show staining of different biomarkers in higher magnification.

## Results

### Histology of the right AVj and RV

To examine the proposed progenitor region, sections of the right AVj were stained with hematoxylin–eosin (Fig. 1A) and Picric Sirius red (Fig. 1D). The anatomical regions represented in the AVj biopsies were the myocardium of the RA and RV, the connective junction between them, and the tricuspid valve. Islands of small cardiomyocytes at the myocardial borders were surrounded by connective tissue (Fig. 1B). Sections from the RV (Fig. 1C) show densely packed, large cardiomyocytes compared with the sparse distribution of the cardiomyocytes near the myocardium border in the AVj. The ECM in the AVj region was rich with high amount of collagen (Fig. 1D). In addition, less collagen was observed in the RV compared with the AVj (Fig. 1E, F).

### Gene expression in the AVj compared with the atria/ventricle

**FIG. 2.**
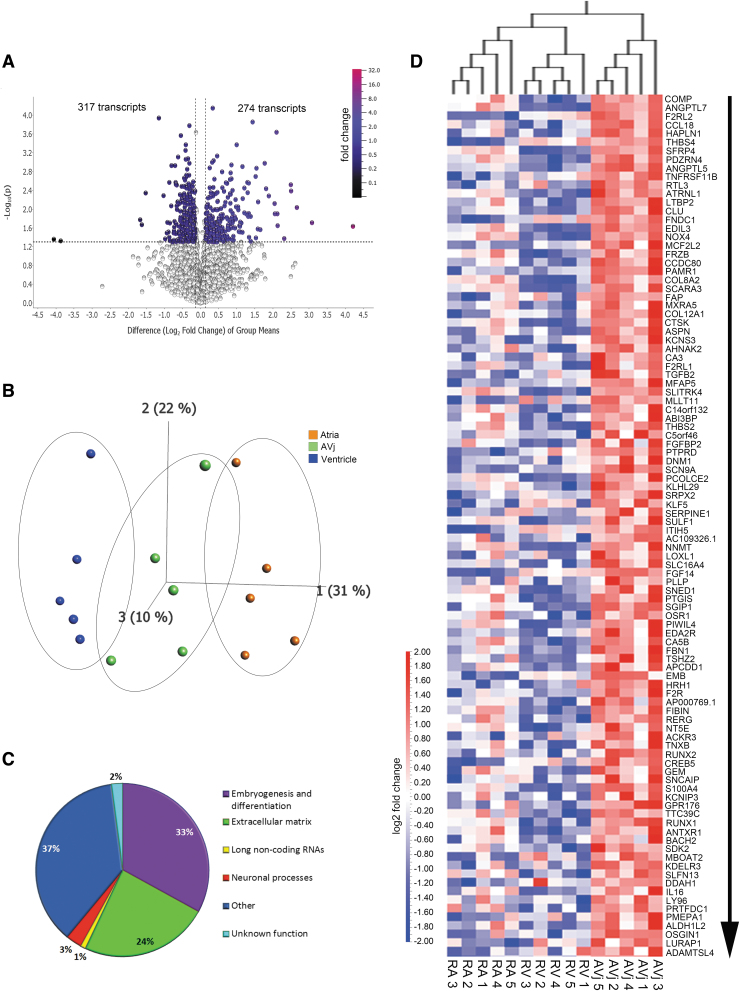
The gene expression analysis of the AVj, RA, and RV was performed using RNA sequencing followed by paired group comparison of five individuals. **(A)** Volcano plot displaying the difference in gene expression between the AVj region and the RA+RV (combined) (log2-fold change, *P* value <0.05). **(B)** Principal component analysis score plot (first three components) of the total number of transcripts detected, showing a separation between the AVj (*green*), atria, (*orange*), and ventricle (*blue*) with regard to gene expression. **(C)** Pie diagram showing the proportion of transcript categories for the 100 most upregulated transcripts in the AVj compared with the RA+RV. **(D)** Heat map of the 100 most upregulated transcripts in the AVj region compared with the RA+RV from five individuals (log2-fold change, *P* value <0.05). The *arrow* indicates the order of the fold change difference (largest to the smallest) of the top 100 most upregulated transcripts.

RNA was isolated from three different regions of the same heart (*N* = 5), sequenced, and analyzed. RNA isolated from the same individual was treated as paired samples. 12 683 transcripts were detected in the gene data set, of which 591 showed altered expression in the AVj area compared with the RV+RA (combined) with *P* < 0.05, although adjusted *P* values did not reach significance (Fig. 2A). Using principal component analysis, an unbiased multivariate classification model, we confirmed a separation between the AVj, RA, and RV with regard to gene expression (Fig. 2B). Focusing on the 100 most upregulated transcripts in the AVj versus the RA and RV (combined), we found that the majority of the transcripts were genes associated with embryogenesis and differentiation (33%), including SFRP4, RTL3, ATRNL1, FRZB, PAMR1, and CtsK, followed by extracellular matrix components (24%) eg, COMP, HAPLN1, THBS4, CCDC80 and COL8A2 (Fig. 2C, D). Additionally, ANGPTL7, a gene involved in negative regulation of angiogenesis, was among the most highly upregulated in the AVj (Fig. 2D).

To obtain more comprehensive insight, we sought to identify altered pathways by performing GSEA on our gene data. The GSEA revealed 36 gene sets that differed between the AVj and RA/RV (Table 1 and Supplementary Table S3). These pathways were all upregulated in the AVj region compared with the RA/RV regions, and the majority was related to cell differentiation and targets associated with tumorigenesis.

**Table 1.** Gene Set Enrichment Analysis

Name	Normalized enrichment score	q
NEWMAN_ERCC6_TARGETS_DN	2.12	0.0226
MEISSNER_BRAIN_HCP_WITH_H3K27ME3	2.02	0.0683
TURASHVILI_BREAST_LOBULAR_CARCINOMA_VS_LOBULAR_NORMAL_DN	1.93	0.0950
HASLINGER_B_CLL_WITH_13Q14_DELETION	1.95	0.0963
chr4p14	1.93	0.1070
CHIANG_LIVER_CANCER_SUBCLASS_INTERFERON_UP	1.96	0.1124
TURASHVILI_BREAST_DUCTAL_CARCINOMA_VS_DUCTAL_NORMAL_UP	1.90	0.1244
NOUSHMEHR_GBM_SILENCED_BY_METHYLATION	1.85	0.1607
BROWNE_HCMV_INFECTION_20HR_DN	1.86	0.1651
HARRIS_BRAIN_CANCER_PROGENITORS	1.87	0.1676
SMID_BREAST_CANCER_RELAPSE_IN_BRAIN_UP	1.86	0.1718
RICKMAN_TUMOR_DIFFERENTIATED_MODERATELY_VS_POORLY_UP	1.83	0.1777
WU_HBX_TARGETS_1_DN	1.80	0.1816
SENESE_HDAC1_AND_HDAC2_TARGETS_DN	1.83	0.1817
GRANDVAUX_IRF3_TARGETS_DN	1.81	0.1819
ONDER_CDH1_TARGETS_2_UP	1.80	0.1845
CHIANG_LIVER_CANCER_SUBCLASS_POLYSOMY7_DN	1.84	0.1847
CERVERA_SDHB_TARGETS_2	1.81	0.1852
TURASHVILI_BREAST_LOBULAR_CARCINOMA_VS_DUCTAL_NORMAL_UP	1.82	0.1917
RTTTNNNYTGGM_UNKNOWN	1.81	0.1919
PAPASPYRIDONOS_UNSTABLE_ATEROSCLEROTIC_PLAQUE_DN	1.79	0.1928
KEGG_ECM_RECEPTOR_INTERACTION	1.78	0.1975
JAEGER_METASTASIS_UP	1.77	0.1979
CERIBELLI_GENES_INACTIVE_AND_BOUND_BY_NFY	1.78	0.1993
KANG_IMMORTALIZED_BY_TERT_DN	1.77	0.2006
MIKKELSEN_IPS_ICP_WITH_H3K27ME3	1.79	0.2009
IZADPANAH_STEM_CELL_ADIPOSE_VS_BONE_DN	1.76	0.2021
BROWNE_HCMV_INFECTION_24HR_DN	1.82	0.2032
BRUECKNER_TARGETS_OF_MIRLET7A3_DN	1.76	0.2037
KONDO_EZH2_TARGETS	1.75	0.2047
FARMER_BREAST_CANCER_CLUSTER_4	1.75	0.2060
INGRAM_SHH_TARGETS_UP	1.77	0.2069
VARELA_ZMPSTE24_TARGETS_DN	1.76	0.2096
JIANG_TIP30_TARGETS_UP	1.76	0.2097
CLASPER_LYMPHATIC_VESSELS_DURING_METASTASIS_DN	1.77	0.2122
HAN_JNK_SINGALING_UP	1.73	0.2483

Pathways differentially regulated in the atrioventricular junction compared with the right atria+right ventricle (combined). Sorted by adjusted *P* value (*q* value) (*q* < 0.25).

### Expression of stem cell niche-related biomarkers in the AVj

Using IHC, tissue sections from eight donors (Supplementary Table S1) were examined for expression of biomarkers associated with stem cell niche biology. Antibodies toward Side population progenitor biomarker MDR1, early cardiac stem cell markers SSEA4, WT1, and hypoxia marker Hif1α were combined with cTnT to distinguish cardiomyocytes and study colocalization. The expression was compared between the proposed progenitor region in the AVj and RV tissue from the same hearts.

**FIG. 3.**
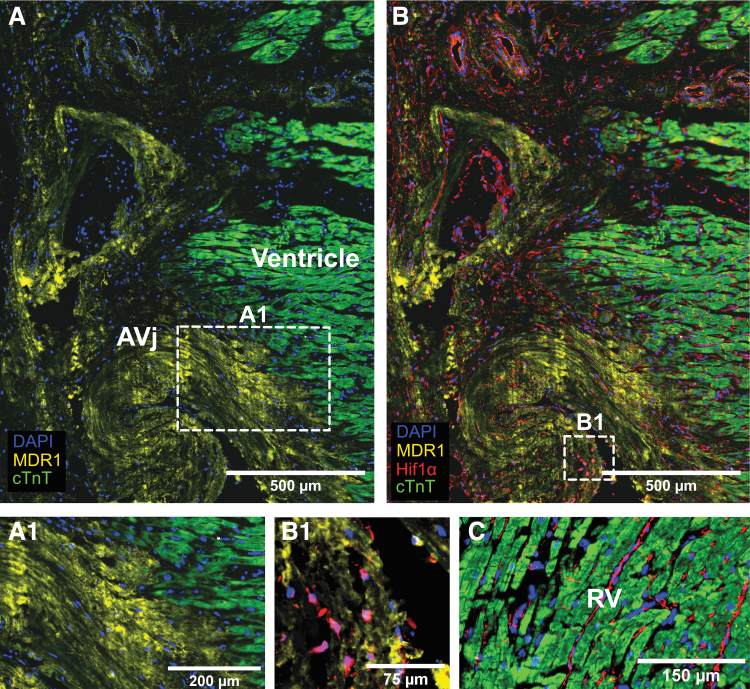
IHC showing the expression of cardiac stem cell marker MDR1 in *yellow*, hypoxia marker Hif1α in *red*, cardiomyocyte marker cTnT in *green*, and nuclei in *blue* in tissue from a representative donor. Vertical stripes are artifacts from the sectioning **(A)**; MDR1 expression was detected in the AVj, at the base of the tricuspid valve. Enlargement of the border between the MDR1+ area and the cardiomyocytes at the edge of the RV, shown in **(A1)**, visualizes a clear difference between cell phenotypes in this area, with minimal gap between MDR1+ and cTnT+ cells. **(B)** Subpopulation of nuclei within the MDR1+ region shows a nuclear staining of Hif1α, indicating hypoxia in the connective tissue of the AVj. Enlargement of a cluster of such MRD1+/Hif1α+ cells is shown in **(B1)**. For comparison, tissue from the RV is shown in **(C)** where no expression of MDR1 or nuclear staining of Hif1α was detected. Cytoplasmatic expression pattern of Hif1α, not related to hypoxia, was observed in the RV. cTnT, cardiac Troponin T; IHC, immunohistochemistry.

Expression of MDR1, a membrane bound pump protein, was detected in large areas (Fig. 3A) where hundreds of cells were stained in tissue from all eight individuals. The cardiomyocytes in the AVj (Fig. 3A1) or in the RV tissue (Fig. 3C) did not express MDR1. When co-stained with Hif1α, a biomarker with nuclear expression in cells under hypoxic conditions, clusters of Hif1α+ nuclei were found within the MDR1+ regions (Fig. 3B, B1) in the AVj. No nuclear expression of Hif1α was observed in myocardium of the AVj biopsies or in the RV. Cytoplasmic expression of Hif1α was observed throughout the tissue by cells between cardiomyocytes (Fig. 3C).

**FIG. 4.**
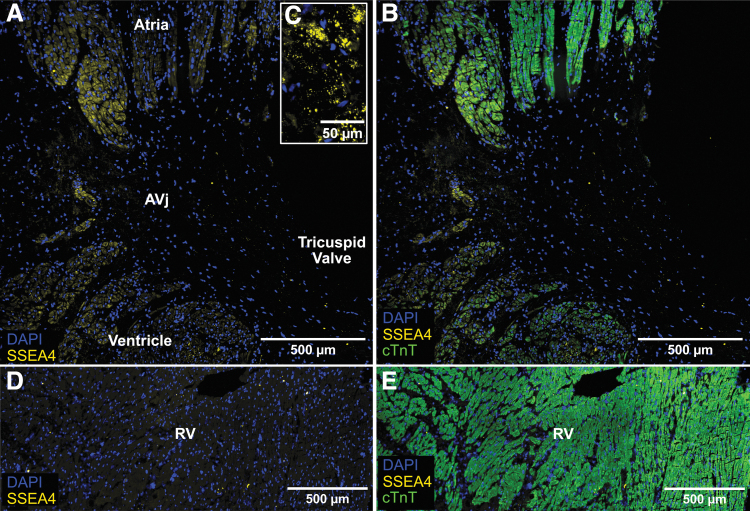
IHC showing expression of stem cell marker SSEA4 in *yellow*, cardiomyocyte marker cTnT in *green*, and nuclei in *blue*. The AVj from one of the representative donors is shown in **(A, B)**. SSEA4 was expressed in cardiomyocytes at the myocardium border of the AVj, with a strong intensity of the staining close to the junction and a weaker signal further into the atrium and ventricle. **(C)** Enlargement of the SSEA4+ cells from another representative donor showing the expression pattern of this glycolipid on cell surface. Images **(D, E)** from a representative RV biopsy show lack of SSEA4 expression.

Cardiac stem cell biomarker SSEA4, a membrane bound glycolipid, was clearly expressed in the AVj (Fig. 4A, C). Unlike MDR1 and Hif1α, SSEA4 was co-expressed with cTnT suggesting expression within the cardiomyocytes at both the atrial and ventricular myocardium borders in the AVj (Fig. 4B). The signal from SSEA4+ cells decreased with the distance from the AVj and was not detected in the RV (Fig. 4D, E).

**FIG. 5.**
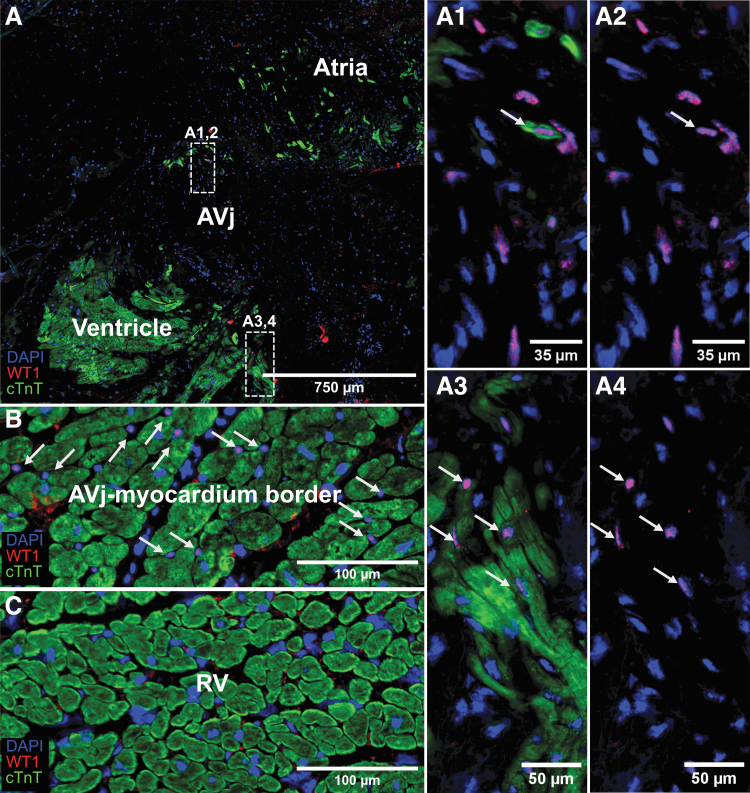
IHC showing expression of cardiac stem cell marker WT1 in *red*, cardiomyocyte marker cTnT in *green*, and nuclei in *blue*. **(A)** WT1 expression in cell nuclei was detected in the AVj region. Enlargement of the upper *boxed* region **(A1,2)** shows one WT1+/cTnT+ cell among WT1+/cTnT− cells. **(A3,4)** The lower *boxed* region at the border of the myocardium of the ventricle. WT1+/cTnT+ cells are highlighted with *arrows*. **(B)** WT1 expression was detected in cells located between the cTnT expressing cardiomyocytes (*arrows*) in the myocardium border of the AVj. **(C)** There was no expression of the WT1 in the RV.

Another embryonic cardiac stem cell marker, the transcription factor WT1, was detected in the nuclei of cells in the AVj. There were also WT1+ nuclei surrounded by a small cTnT+ cytoplasm, suggesting early cardiomyocytes (Fig. 5A1–4). More commonly was the finding of WT1+ cells between cardiomyocytes in the atrial and ventricular myocardium borders of the AVj (Fig. 5B). WT1 was not expressed in the RV (Fig. 5C).

**FIG. 6.**
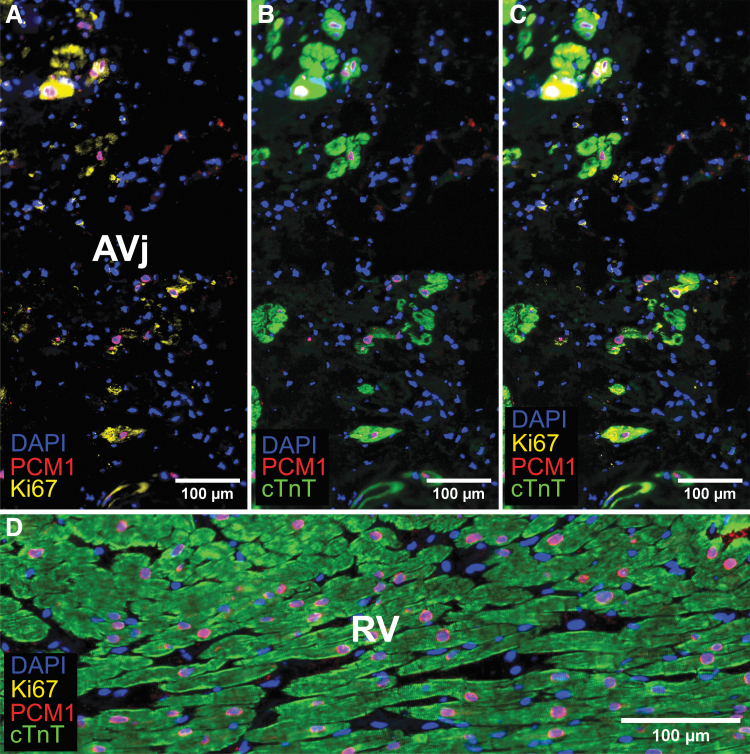
IHC of cardiac tissue at the AVj region with proliferation-related marker Ki67 in *yellow*, cardiomyocyte nuclei marker PCM1 in *red*, sarcomeric marker cTnT in *green*, and nuclei in *blue*. **(A–C)** The figure shows the diversity among the cardiomyocyte subpopulations within the AVj region; small PCM1+/cTnT− cells were found, indicating immature cardiomyocytes. Additionally, expression of Ki67 was found in rare cells, both PCM1+/cTnT+ cells and PCM1+/cTnT− cells, indicating cycling cardiomyocytes during various stages of maturity. **(D)** Neither PCM1+/cTnT− cells or Ki67+ nuclei could not be found in the right ventricular tissue.

**FIG. 7.**
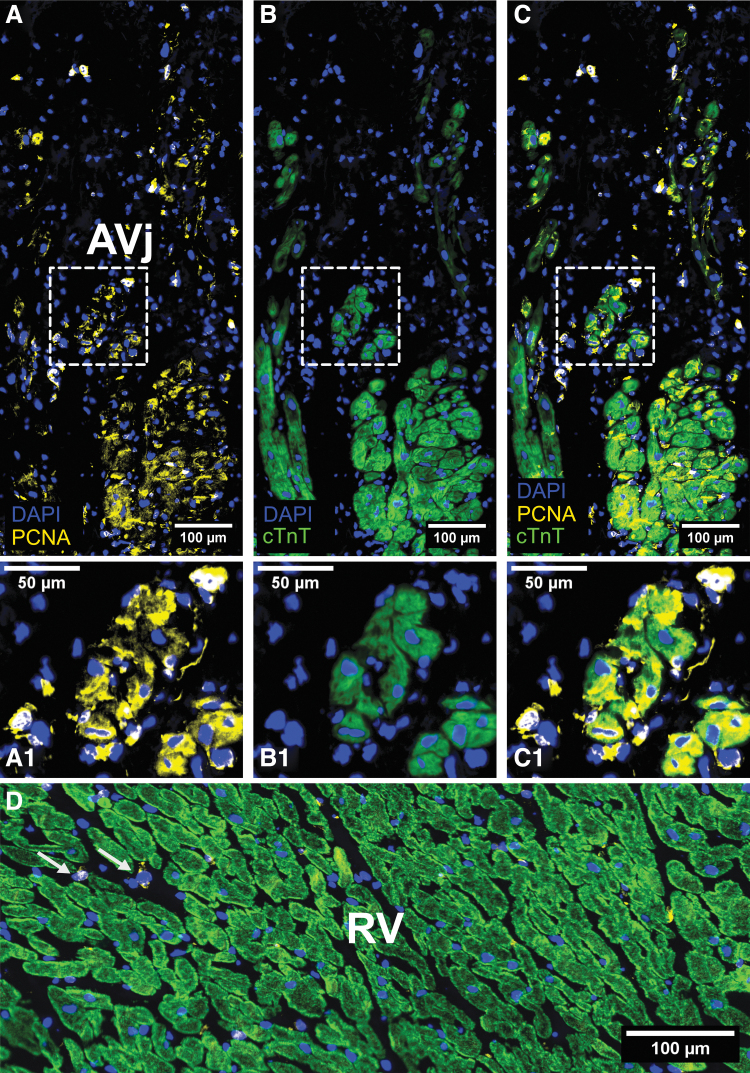
IHC with proliferation-related marker PCNA in *yellow*, cTnT in *green*, and nuclei in *blue*. **(A–C)** Expression of PCNA was found in many cells in the AVj, occasionally colocalizing with cTnT surrounding the cytoplasm indicating proliferation of cardiomyocytes. These PCNA+/cTnT+ cells were only found in the border to myocardium. **(D)** PCNA+ cells could be found in the right ventricular myocardium between the cardiomyocytes (*arrows*). PCNA, proliferating cell nuclear antigen.

Additionally, the proliferation marker Ki67 was expressed by rare cell nuclei in the AVj region (Fig. 6). To determine whether the Ki67 expressing cells were cardiomyocytes, the specific cardiomyocyte nuclei marker PCM1 was used. Interestingly, few Ki67+/PCM1+ nuclei were found with weak signal for cTnT (Fig. 6A–C). These cells, as well as few PCM1+/cTnT− cells, were interpreted as early cardiomyocytes. Since we could not find the Ki67+ cells in tissue from several donors, we used another proliferation-related marker, PCNA. Many more PCNA+ cells were found compared with Ki67 in the AVj from all donors. Most of the PCNA+ cells were cTnT−. However, we did find PCNA+/cTnT+ small cardiomyocytes only in the border to myocardium in the AVj (Fig. 7A–C). PCNA expression was observed in cTnT− cells in the RV (Fig. 7D) where no expression of Ki67 could be detected (Fig. 6D).

Taken together, a diversity among the cell subpopulations in the AVj was shown ranging from progenitors to early cardiomyocytes. Among the progenitors, there were MDR1+, SSEA4+, and WT1+. Among the early cardiomyocytes, there were SSEA4+/cTnT+, WT1+/cTnT+, and PCM1+/Ki67+ cells with small cytoplasm as well as the PCM1+/cTnT− cells.

## Discussion

Cardiac progenitors could be used in therapies for cardiovascular disease, such as heart failure where loss of cardiomyocytes is the main issue. Most previous studies in the field of cardiac stem cells have been based on small animal models, which may not reflect the human setting. We have analyzed freshly frozen tissue from human hearts, which makes our results clinically relevant and important in the pursuit of finding the origins of cardiac regeneration.

In the human heart, the search for a stem cell niche is still ongoing. Kimura et al. presented small, hypoxic, proliferative cardiomyocytes in mice using lineage tracing and hypoxia [26]. Previously, we identified a potential stem cell niche in the AVj of the adult rat heart around the tricuspid and mitral valves [17]. In a follow-up study, we analyzed the human left AVj, the base of the mitral valve, and reported expression of biomarkers associated with hypoxia, stem cells, proliferation, and migration [18]. In the present study, we explored the base of the tricuspid valve. Extensive characterization with RNA sequencing and IHC was performed since DNA labeling is not possible in humans.

The AVj region consists of the annulus fibrosus at the base of the valve leaflets, which are derived from proepicardium during development [27]. This junction plays an important role as physical barrier between the atrial and ventricular myocardium. Our histological analysis showed that the AVj is rich in collagens. Composition of ECM provides an optimal milieu and keeps stem cells in an undifferentiated state [8].

In support of the histology data, we observed a similar pattern when analyzing gene expression in the AVj compared with the RA/RV. The most upregulated protein-coding genes in the AVj were associated with embryogenic and developmental processes as well as ECM components and regulation of ECM. The GSEA of the gene data revealed a similar pattern of upregulated pathways associated with cell differentiation in the AVj region compared with the RA/RV. The extent and specificity regarding the differential gene expression between the AVj region and RA/RV need to be confirmed with more precise methods since the adjusted *P* values failed to reach significance.

The AVj biopsies, free dissected by hand, likely contain parts of atria and ventricle, which might explain the unsatisfactory statistics. We have previously tried spatial transcriptomics that has been optimized for cardiac tissue [28], but the high collagen content in the AVj created artifacts and challenged the readout. However, combined with the histological data, there is a clear indication of a progenitor activity and diversity within the ECM composition in the AVj.

Hypoxia is one of the key factors for maintaining the quiescent of stem cells in the niches, as shown in the bone marrow [29]. Low oxygen microenvironment was shown in the epicardium by quantification of nuclear HIF1α expression and capillary density [30]. Important findings in the present study are the nuclear expression of HIF1α in collagen-rich region of the AVj as well as the upregulation of ANGPTL7, a gene involved in negative regulation of angiogenesis. Furthermore, expression of HIF1α overlapped with the MDR1+ cells, connecting this stem cell marker to the hypoxic region. Previously, we reported the absence of CD31+ endothelial cells in the streak of MDR1+ cells indicating hypoxia in the left AVj [18]. MDR1 is a cardiac-specific stem cell marker, associated with Side population cells, as shown in the mouse [31] and human heart [22]. Our finding of these MDR1+/cTnT− cells in the AVj shows that these progenitors are not committed to the cardiomyocyte lineage but rather of more immature phenotype.

Another early cardiac marker SSEA4 has previously been identified in the adult human [18,20] and sheep heart [32]. We found hundreds of SSEA4+ cells in the AVj myocardium in tissue from all included donors. Interestingly, an SSEA4+/cTnT+ signature of these cells suggests that SSEA4 is a cardiomyocyte-specific progenitor marker in the adult human heart. Recently, SSEA4+ cells were described as immature cycling cardiomyocytes in the adult human heart, in line with our finding [33].

WT1 is involved in the epicardial epithelial–mesenchymal transition, as well as the formation of the coronal vasculature system during the development [34]. Throughout adulthood, WT1 continues to be expressed in the heart vasculature [21,35]. This is mostly in line with our findings, where WT1+/cTnT− cells between the cardiomyocytes, suggesting capillaries in the myocardium border of the AVj. However, WT1+ small cTnT+ cardiomyocytes in the AVj region imply that WT1 might be involved in cardiomyocyte formation as well and mirror our previous finding in the left AVj [18].

Small cardiomyocytes were only present at the border to the myocardium in the AVj, compared with the RV. As PCM1 is specific for cardiomyocyte nuclei, PCM1+/cTnT− cells were interpreted as another early stage in cardiomyocyte development. Furthermore, the AVj was the most active site for proliferation markers PCNA and Ki67. Rare Ki67+/PCM1+/cTnT+ cells could be found, indicating proliferative capacity of a rare cardiomyocytes.

In our search for the origin of cardiac regeneration, we found slow cycling cells around the mitral and tricuspid valves in the murine hearts [17]. The results from the present study on the right AVj mirror our earlier findings from the left AVj [18] showing the same pattern, the ECM-rich region with large streaks of MDR1+ cells, and nuclear expression of HIF1α indicating hypoxia. The decreasing SSEA4 expression with distance from the AVj was observed on both sides of the human heart in line with the decrease in intensity of the BrdU staining with the distance from the mitral valve in the murine hearts, that we quantified [17]. This phenomenon might be interpreted as a differentiation and a gradual maturation of the cardiomyocytes.

An ultimate stem cell identifier may not exist. Thus, future studies are needed to investigate the function of the cells identified here either by isolation of viable progenitors and in vitro differentiation into beating cardiomyocytes or by lineage tracing studies in animal models. Another limitation of the study is the low number of included individuals and the wide range of age and medical history making it difficult to draw conclusion based on the clinical background.

## Conclusions

We explored the right AVj for expression of stem cell niche-related biomarkers. Co-expression of early cardiac stem cell biomarkers SSEA4+and WT1+ and the Side population marker MDR1+ as well as the hypoxia marker HIF1α, all in the same anatomical region, suggests that the right AVj harbors a stem cell niche, similarly as we previously reported from the left AVj. The observation of SSEA4+/cTnT+, WT1+/cTnT+ cells shows cardiomyocyte lineage commitment. Finding cTnT+ small cardiomyocytes, co-expressing proliferation markers PCNA and Ki67, as well as the PCM1+/cTnT−, point toward the generation of immature cardiomyocytes. The identification of a novel anatomical site with immature cardiomyocyte populations opens vast opportunities for improving endogenous regeneration.

## Supplementary Material

Supplemental data

Supplemental data

Supplemental data
