# Supplementary material for: Expression of Stem Cell Niche-Related Biomarkers at the Base of the Human Tricuspid Valve
Source: Stem Cells Dev. 2023 Mar 3;32(5-6):140–51. doi: 10.1089/scd.2022.0253 (PMC9986114; doi:10.1089/scd.2022.0253)
Supplement: Supplemental data [file Suppl_TableS1.docx]

**Supplementary Table 1: Clinical background for donors**

| Donor | Gender | Age | | Cause of death | Medical history | | Tissue used for | | |
| --- | --- | --- | --- | --- | --- | --- | --- | --- | --- |
| 1 | F^1^ | 63 | Ischemic cerebral edema, due to cardiac arrest | | | Ischemic heart disease, hypertension, obesity, hypothyroidism, diabetes type 2, renal insufficiency, emphysema | | | RNAseq^6^ |
| 2 | F | 42 | Intracerebral hemorrhage | | | Takotsubo cardiomyopathy in the acute setting | | | RNAseq |
| 3 | M^2^ | 52 | Cardiac arrest | | | HF^4^ in the acute setting | | | RNAseq |
| 4 | F | 75 | Intracerebral haemorrhage | | | Atrial Fibrillation, ischemic  heart disease, previous AMI | | | RNAseq |
| 5 | M | 74 | Intracerebral hemorrhage | | | Previous stroke | | RNAseq, IHC^7^ | |
| 6 | F | 31 | Brain tumour | | | None | | | IHC |
| 7 | M | 62 | Subarachnoid haemorrhage | | | Atrial fibrillation, maze surgery | | | IHC |
| 8 | F | 50 | Intracerebral haemorrhage | | | Previous Ventricular Tachycardia, suspected previous AMI^3^, suspected Takotsubo cardiomyopathy | | | IHC |
| 9 | F | 19 | Ischemic cerebral edema due to cardiac arrest | | | Anorexia | | | IHC |
| 10 | F | 43 | Ischemic cerebral edema due to cardiac arrest | | | None | | | IHC |
| 11 | M | 54 | Subarachnoid haemorrhage | | | Suspected LAD^5^ dissection | | | IHC |
| 12 | M | 69 | Traffic accident | | | None | | | IHC |

Table 1 summarizes the clinical background for the donors not suitable for cardiac transplantation.
^1^Female, ^2^Male, ^3^Acute Myocardial Infarction, ^4^HF = Heart Failure, ^5^Left Anterior Descending Artery, ^6^Ribonucleic Acid Sequencing, ^7^Immunohistochemistry
