# Supplementary material for: Expression of Stem Cell Niche-Related Biomarkers at the Base of the Human Tricuspid Valve
Source: Stem Cells Dev. 2023 Mar 3;32(5-6):140–51. doi: 10.1089/scd.2022.0253 (PMC9986114; doi:10.1089/scd.2022.0253)
Supplement: Supplemental data [file Suppl_TableS2.docx]

**Supplementary Table 2: Antibodies used in immunohistochemistry**

| **Biomarker** | | **Primary antibody** | **Concentration**  **(mg/ml)** | **Dilution** | **Manufacturer** | **Catalogue number** |
| --- | --- | --- | --- | --- | --- | --- |
| **Wt1** | Rabbit monoclonal IgG | | 0.3 | 1:250 | Abcam | ab89901 |
| **SSEA-4** | Mouse monoclonal l gG3 | | 0.5 | 1:50 | eBioscience | 14-8843-80 |
| **MDR1** | Rabbit monoclonal IgG | | 0.3 | 1:250 | Abcam | ab170904 |
| **cTnT** | Mouse monoclonal IgG1 | | 0.2 | 1:50 | Thermo Fisher^1^ | MS-295-P1 |
| **HIF1α** | Mouse monoclonal IgG1 | | 1.0 | 1:300 | Novusbio | NB-100-131 |
| **Ki67**  **PCM1** | Mouse monoclonal IgG1  Rabbit polyclonal IgG | | 0.2  0.3 | 1:200  1:400 | Santa Cruz^2^  Atlas | sc-2390  HPA023374 |
| **PCNA** | Mouse monoclonal IgG2a | | 1.0 | 1:1000 | Abcam | ab-29-100 |

Table 2 summarizes the information about the primary antibodies used for immunohistochemistry. ^1^Thermo Fisher Scientific, ^2^Santa Cruz Biotechnology
